# Experimental platforms to study blast injury

**DOI:** 10.1136/jramc-2018-000966

**Published:** 2018-05-24

**Authors:** Thuy-Tien Nguyen, A P Pearce, D Carpanen, D Sory, G Grigoriadis, N Newell, J Clasper, A Bull, W G Proud, S D Masouros

**Affiliations:** 1 Department of Bioengineering, Imperial College London, London, UK; 2 Academic Department of Military Surgery and Trauma, Royal Centre for Defence Medicine, Birmingham, UK; 3 Institute of Shock Physics, Imperial College London, London, UK; 4 Department of Orthopaedics and Trauma, Frimley Park, Frimley, UK

**Keywords:** blast injury, experiments, interdisciplinary, blast

## Abstract

Injuries sustained due to attacks from explosive weapons are multiple in number, complex in nature, and not well characterised. Blast may cause damage to the human body by the direct effect of overpressure, penetration by highly energised fragments, and blunt trauma by violent displacements of the body. The ability to reproduce the injuries of such insults in a well-controlled fashion is essential in order to understand fully the unique mechanism by which they occur, and design better treatment and protection strategies to alleviate the resulting poor long-term outcomes. This paper reports a range of experimental platforms that have been developed for different blast injury models, their working mechanism, and main applications. These platforms include the shock tube, split-Hopkinson bars, the gas gun, drop towers and bespoke underbody blast simulators.

**Key messages**

- A range of experimental platforms is essential to recreate the various injury mechanisms due to an explosion.
- The versatility of experimental devices ensures their adaptability to specific in vivo, ex vivo, and in vitro experimental models of blast injury.
- Ensuring that the boundary conditions of the sample tested are appropriate and biofidelic is key for validation of computational simulations and for replicating specific blast scenarios and injury mechanisms.
- The relevance, design, fabrication and translation of these experimental platforms requires a wide range of expertise and is dependent on interdisciplinary collaboration between science, engineering, and medicine.

## Introduction

Modern conflict and terrorist attempts in civilian settings have seen an ever-increasing use of explosive devices as the weapon of choice. At the same time, medical treatment at the point of wounding and beyond has reached unprecedented levels of efficacy, resulting in an increased number of survivors with severe injuries.^1^ The epidemiological study by Penn-Barwell *et al* 1 on UK personnel in Iraq and Afghanistan between 2003 and 2012 reported a greater probability of survival for a given injury burden (as defined by the New Injury Severity Score—NISS). A NISS of 75 had less than 2.5% probability of survival in 2003, but approximately 15% in 2012. Blast from explosive weapons was found to be the main injury mechanism in this study (70%). Similarly, a study by Eastridge *et al* 2 on the fatality of the US troops during Operation Iraqi Freedom and the Operation Enduring Freedom between 2001 and 2011 reported that 74% of the lethal injuries were caused by explosive devices. Use of explosive weapons is not exclusive to the military setting as these are increasingly used against civilians in terror attacks such as in the Oklahoma City bombing in 1995, the Atlanta Olympic Park bombing in 1996, the London underground bombing in 2005, the Madrid train attack in 2006, the Boston Marathon bombing in 2013, the New York City and New Jersey twin bombing in 2016, and the Manchester arena bombing in 2017 to name but a few.^3^ The injuries from explosive munitions are very different to those caused from conventional weapons and also less well characterised.^4^

Historically, blast injuries have been categorised mainly based on how the physical aspects of the explosion act in causing the injury.^5^ Primary blast effects are associated with the blast wave. They produce barotraumas, whereby the blast wave reaches and accelerates structures of different densities causing them to displace and to develop stress and shear waves within them. Organs with high air content such as the lung, the bowel, and the middle ear are the most vulnerable to this type of injury. Secondary blast effects are caused by highly energised objects, such as parts of the device casing, purposely added fragments or debris from the vicinity of the device, which are carried by the wake of the blast wave. These may reach the human body at very high speeds, resulting in ballistic-type penetrating injuries to soft and skeletal tissue. Large-scale disruptive injuries such as traumatic amputation are likely to be due to the combination of primary and secondary blast effects. Tertiary blast effects describe the blunt impact and crush injuries that result from blast-induced displacement of personnel, or hard objects. Quaternary blast injuries are associated with burns, inhalation of toxic gases, or environmental contamination, and quinary blast injuries are due to hyperinflammatory behaviours possibly due to toxins added as unconventional contents in the explosive.^6^ In addition, post-traumatic complications such as heterotopic ossification (HO), neurotrauma, stress disorders, and recurrent infections may be significant, long-term sequela for blast casualties. These effects currently are not well understood, but there is substantial international research effort employed to do so.

To understand blast injury in depth, it is important to decouple the various injury mechanisms and to recreate time-dependent effects of blast in well-controlled laboratory environments. An explosion is a dynamic process that evolves with time. Detonations can compress the surrounding media to hundreds of kilobar inside the fire ball; the pressure quickly drops to a few tens of bar just outside the fire ball and exponentially decays to the ambient pressure with distance.^7^ The energy produced from an explosion can accelerate small fragments to more than 1000 m/s, which then decelerate with distance, nominally to less than 600 m/s when striking human victims.^8^ This energy can also accelerate the human body, entirely or partially, to tens of metres per second in a matter of milliseconds.^9^ These mechanical loadings vary greatly depending on the size and type of the explosive source, the distance of the victim from it, the surrounding environment (free field, in vehicle, urban area, buried with wet or dry soils), and the specific interaction with victims (mass, orientation, number of exposures).

Due to this complicated nature of explosions, the resulting trauma is usually due to multiple types of injury mechanism, rendering the design of protective strategies and of ongoing casualty care rather challenging. Research studies on blast injury require experimental capability outside the loading range that most conventional machines can offer and with a versatile tunability for individual parameters of the simulated blast. This paper presents and discusses the development and application of a range of platforms that can satisfy the dynamic conditions of blast loading, and focuses on platforms developed in the Royal British Legion Centre for Blast Injury Studies (CBIS) at Imperial College London.

## Primary blast effects—the shock tube and split-Hopkinson pressure bar

### Shock tube

**Figure 1:**
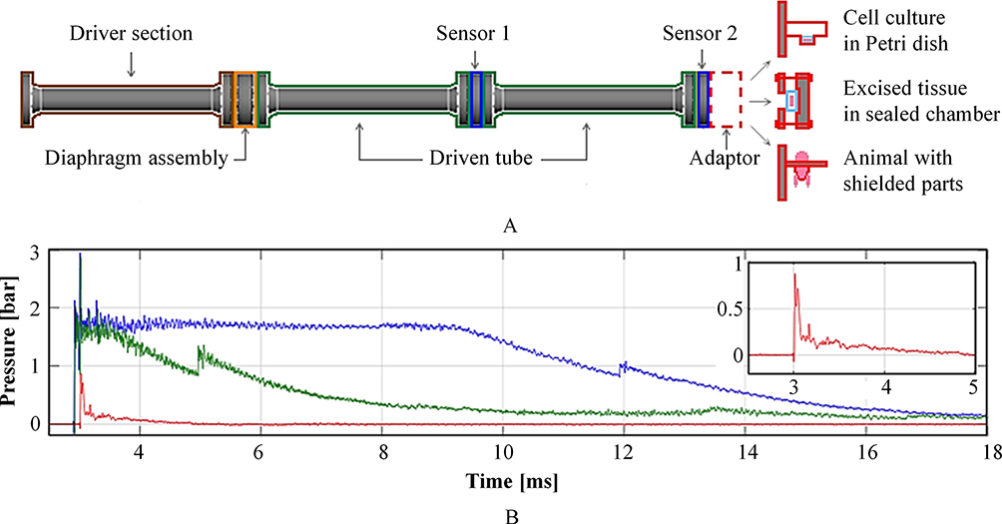
(A) Schematic of the CBIS shock tube with adaptors for in vitro, ex vivo, and in vivo studies. (B) Examples of different blast loading profiles produced by the shock tube, with blow-out of the open-air Friedlander waveform.

The shock tube is a conventional apparatus for generating the pressure profile of the blast wave as it is transmitted away from the fire ball region of the explosion. The CBIS shock tube is a stainless steel, 3.8 m long, air-driven system with 59±1 mm internal bore (Figure 1A). The system is designed to replicate the blast loading conditions of various explosion scenarios, such as open-field air blast (Friedlander waveform), partially confined blast and fully confined blast, with magnitude between 0.5 and 10 bar (Figure 1B).^10^ The driver section is pressurised with compressed air to the required firing pressure, which is regulated by diaphragms in the diaphragm assembly, while the driven section remains at atmospheric pressure. As the burst pressure is reached, the rupture of diaphragms generates a blast wave that propagates along the driven section and subsequently reaches the studied sample at the end of the shock tube. The diaphragm thickness, length of firing air volume, and inserted structure such as perforated plates and granular beds can be used to tailor the pressure loading profile to a desired blast scenario.

At the target end, the system can be adapted to study the effect of blast loading on biological specimens such as in vitro cell cultures, ex vivo tissues/organs, and in vivo whole animal models (Figure 1A). Eftaxiopoulou *et al* used a platform where only the left limb of the rodent specimen was exposed to the blast wave to avoid unwanted complication in other body parts.^11^ The study found that regardless of peak pressure magnitude, 5-millisecond-duration blasts can cause acute inflammation, but 3-millisecond-duration ones cannot. Arora *et al* 12 performed whole animal model experiments to investigate microstructural changes in rodent lungs after exposure to a blast wave. Open-air blast loadings of two different pressure magnitudes were used in which only the higher pressure level resulted in zones of significant focal injuries. Other examples include experimental models using excised rodent lungs and ex vivo organ cultures of porcine respiratory tissue for studies of primary blast-lung injuries,^10^ and in vitro cell cultures for identifying the pathology behind HO^13 14^ as well as an in vitro rodent organotypic brain-slice culture to assess the treatment of blast-related traumatic brain injury with xenon gas.^15^

### The split-Hopkinson pressure bar (SHPB)

**Figure 2:**
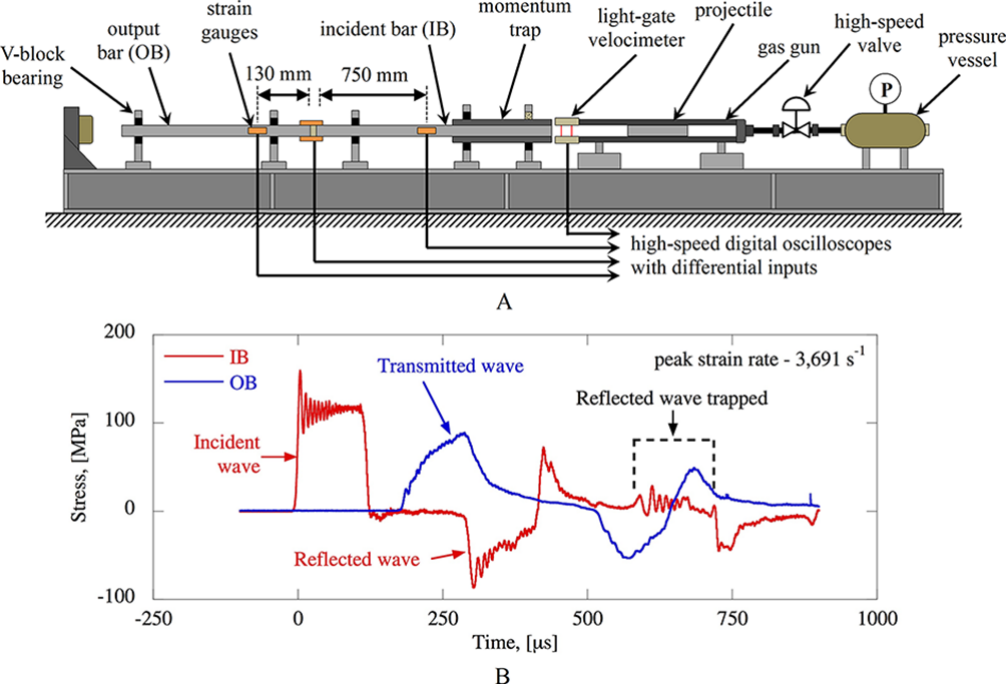
(A) Schematic of the modified split-Hopkinson pressure bar (SHPB) system. (B) Example of loading pulse on the SHPB. The momentum capture system traps the reflected wave and prevents the sample from being loaded multiple times.

The SHPB is a versatile, mechanical loading apparatus used to generate loadings over time durations from hundreds of microseconds up to one millisecond, therefore achieving strain rates in the hundreds and thousands per second. A conventional SHPB system consists of an arrangement in series of two long, cylindrical bars called the input bar (IB) and the output bar (OB) between which the sample of interest is sandwiched (Figure 2). A projectile bar impacts the free end of the IB thus generating a longitudinal compressive stress wave that travels through the system. The loading conditions at the sample are inferred from the strain–time histories recorded on the bars using the principles of one-dimensional elastic wave propagation and can be tweaked by adapting the firing pressure of the projectile bar. On the CBIS SHPB system, a momentum trap is fitted on the IB to ensure sample recovery after the completion of the first loading event.

Based on a modified SHPB apparatus, Sory *et al* 16 17 developed an in vitro platform that incorporates the features of three-dimensional (3D) cell cultures under blast loading conditions. The platform consists of a biocompatible pressurisation chamber used to accommodate the 3D cell-seeded scaffolds under sterile conditions. Central to the development of the system was the definition of reproducible compressive loading histories featured with experimentally quantifiable mechanical parameters.

The platform has also been used to examine the effects of blast-relevant mechanical insults on the stimulation of osteogenesis in periosteum-derived mesenchymal stromal cells (PO MSCs) encapsulated in a 3D scaffold.^17 18^ Mechanically stimulated osteogenesis in PO MSCs was achieved, quantified through the upregulation of key osteogenic markers, including Runx2 and Osteocalcin genes. The stimulation of osteogenesis in MSCs was shown to occur due to the combined action of several mechanical parameters, suggesting that cells are finely tuned to respond to mechanical stimuli that fall within defined ranges of strain rates, stress, and impulse.

## Secondary blast effects—the gas gun

**Figure 3:**
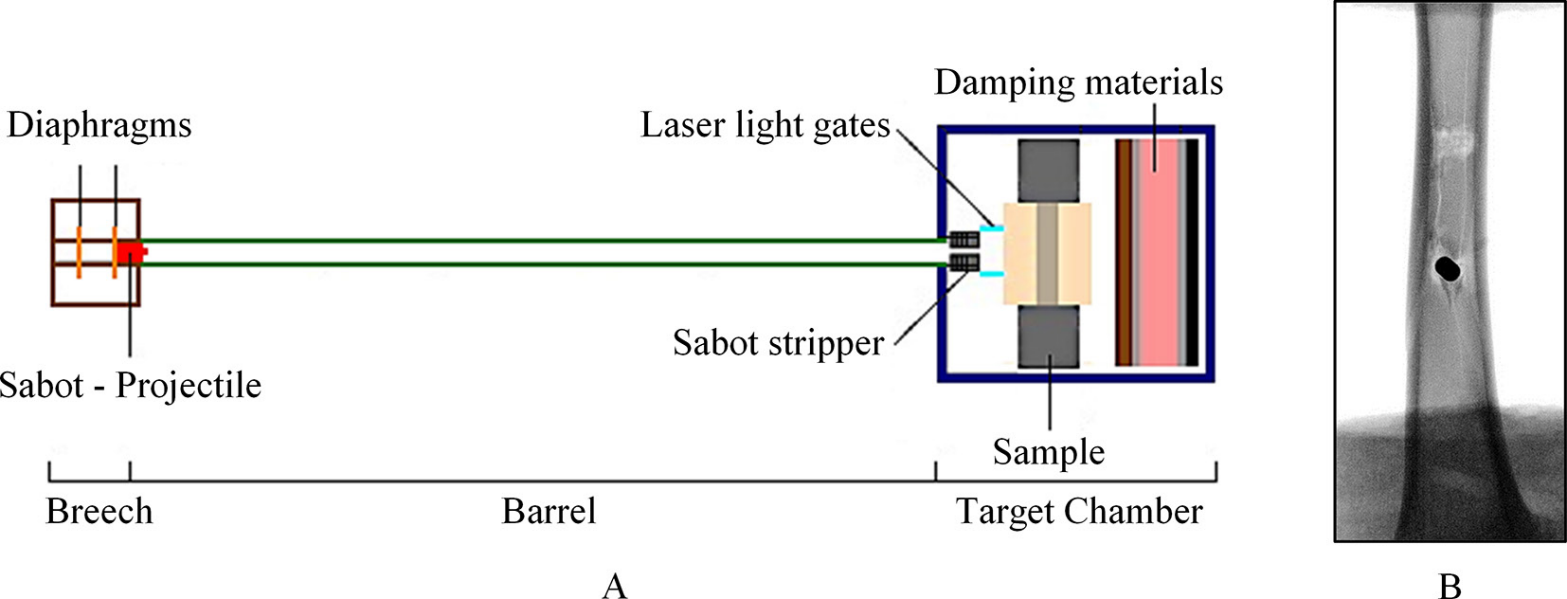
(A) Schematic of the CBIS gas-gun system with target chamber set-up for fragment penetration to the tibia. (B) Radiograph of an ovine tibia penetrated by a projectile at the anterior surface producing a fragmented-wedge fracture pattern.

A 32-millimetre-bore gas-gun system has been developed to investigate secondary blast-injury mechanisms (Figure 3A).^19^ The gas gun consists of a breech section that can be charged up to 200 bar-litre with either compressed air or helium. The double-diaphragm system is used as the firing and controlling mechanism where the prime section between the two diaphragms is charged to below the diaphragm burst pressure and the reservoir section behind the first diaphragm is charged to twice that value. As the prime pressure vents to atmosphere, the diaphragms rupture, releasing the high-pressure gas, which accelerates the sabot (which carries the projectile of choice) behind the second diaphragm along the 3-metre-long barrel of the gas gun towards the target chamber. The velocity of the projectile is proportional to the firing pressure in the reservoir section, which in turn relates directly to the thickness of the Mylar diaphragms used. The current gas-gun system can accelerate projectiles up to 600 m/s.

To adapt the system for studies with fragment-simulating projectiles (FSPs) whose dimensions are usually much smaller than the gas-gun bore, the sabot is designed with an aluminium front plate, which can accommodate FSPs of desired shapes and sizes. A sabot stripper construction is installed at the entrance to the target chamber to halt the sabot while allowing the FSP to continue towards the target. Additional safety layers of wood, rubber, and steel are padded at the back of the target chamber to annihilate any remaining kinetic energy of the FSP if it passes through the sample.

This gas-gun system is currently used to investigate the penetration of blast fragments into soft tissue simulants and the tibia. Studies by Nguyen *et al* 19 20 report results from the penetration of cylindrical and spherical FSPs into ballistic gelatine, 20% by weight, acting as the subdermal tissue simulant, and the effect of projectile speed at impact on the type of fracture caused in tibia samples (Figure 3B).

## Tertiary blast effects—RivUL, drop towers, and AnUBIS

### Drop towers

**Figure 4:**
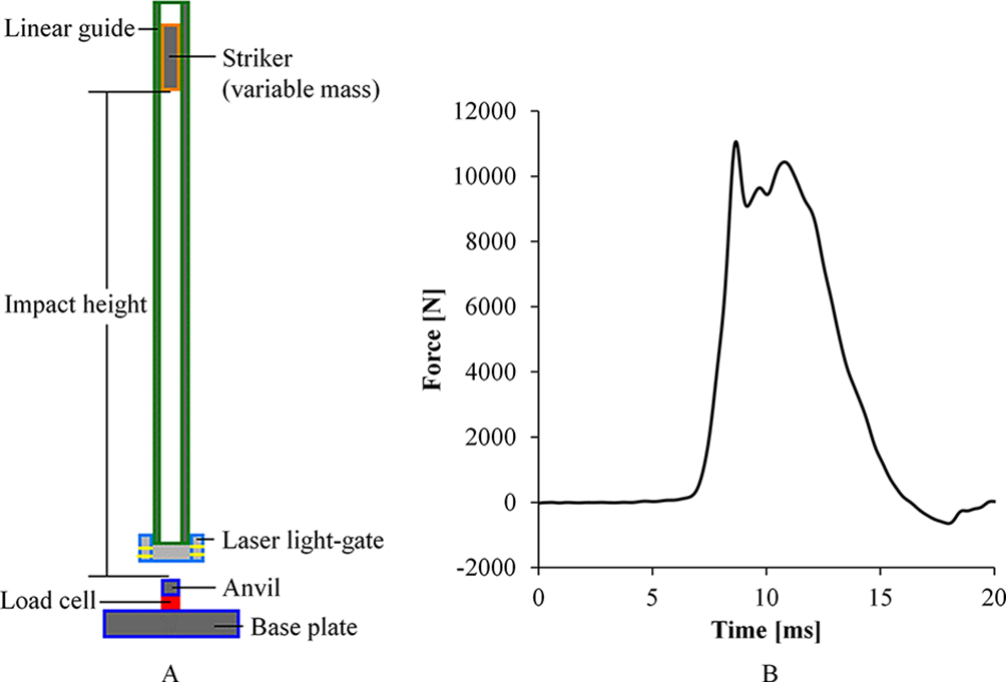
(A) Schematic of the drop tower. (B) Typical force–time response in a pelvic impact test.

The Gardner impact test, commonly known as drop-weight impact test, is characterised by the vertical dropping of an impactor of variable mass, striking a specimen at the base of the tower. This platform can be used to study injuries by blunt impact, characterise tissue at high strain rates, as well as to investigate the performance of protective equipment under impact loading. The impact energy is determined as a function of the drop height and drop mass. Three CBIS drop towers are of similar design (Figure 4A) but vary in size to suit various applications.

Webster^21^ investigated whether axial load alone was adequate to disrupt the pelvis at the pubic symphysis (PS) and the sacroiliac (SI) joints, an injury seen commonly in the dismounted casualty in recent conflicts. Pelvic specimens were tested under axial impact loading through the femur at an energy level of 330 J (Figure 4B). It was found that pure axial load did not produce opening of the PS or SI joints; rather it produced preferential femoral neck fracture rather than pelvic disruption. Sory used a small drop-tower apparatus (0.3 to 2.7 J target energy levels) to investigate the stimulation of osteogenesis in MSC-based engineered samples subject to loading ranging from 100 to 600 s^−1^.^17^

### AnUBIS—Anti-vehicle Underbelly Blast Injury Simulator

**Figure 5:**
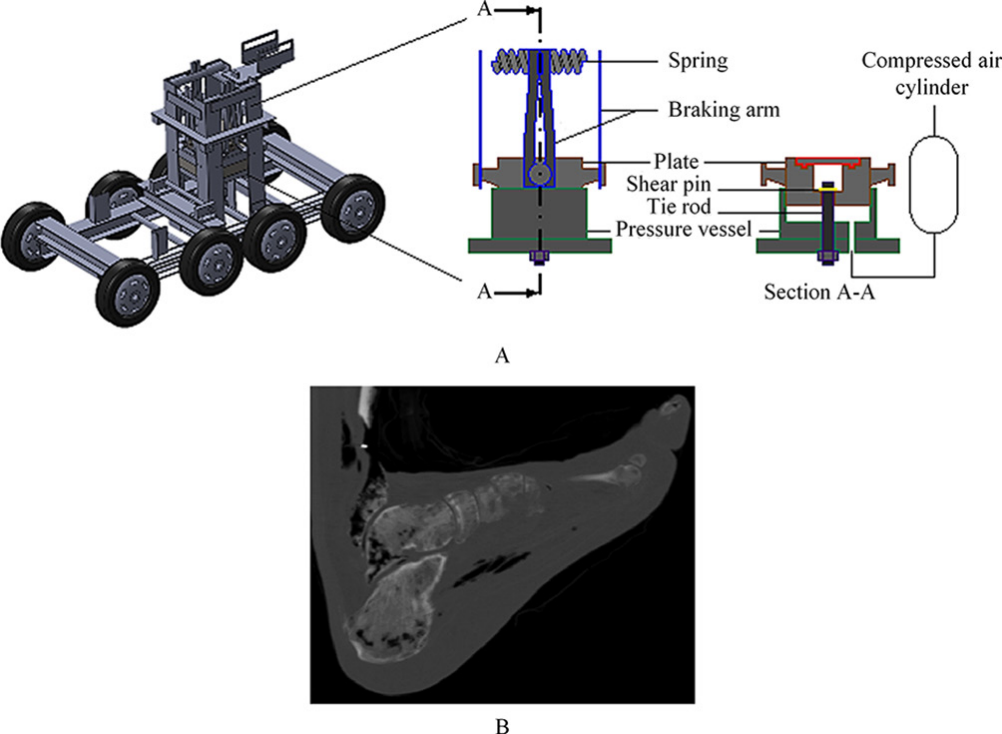
(A) Anti-vehicle Underbelly Blast Injury Simulator (AnUBIS) schematic. (B) Radiograph of a fractured foot produced from AnUBIS loading.

AnUBIS has been developed to replicate the loading applied to occupants’ lower limbs in underbody blast (UBB).^22^ The lower limbs rest on a plate, which is accelerated to a target velocity (up to 20 m/s) within a few millimetres, before rapidly decelerating to rest. The acceleration of the plate is achieved with compressed air at the underside of the plate. The acceleration profile and target velocity are controlled through careful selection of the material and geometry of a pin, which is designed to hold the plate in place until a specific underside pressure is reached, at which point the pin shears (Figure 5A).

AnUBIS has been used in conjunction with postmortem human specimens (PMHSs) and instrumented anthropometric test devices (ATDs) to estimate the probability of injury as well as to assess the performance of protective equipment. Using both PHMSs and the Hybrid-III ATD, Grigoriadis *et al* 23 investigated whether changing the seated posture from 90°−90° alters the injurious outcome (Figure 5B). The study suggests that the force values obtained from the Hybrid-III when its placement deviates from the typical 90°−90° might be misleading if used to assess injury risk in the human lower extremity. Similarly, a standing posture was shown to result in a significantly more severe injury than a seated posture in AnUBIS tests with PMHSs,^24^ suggesting that standing military-vehicle occupants are at higher risk of injury than seated occupants.

### RivUL—Rig for in vivo Underbody Loading

Although AnUBIS is able to recreate the lower limb musculoskeletal injuries caused by UBB, the system (and others like it) uses cadaveric tissue. While this is arguably biofidelic anatomically, it is not reliable for recreation of internal organ injuries, which are important for prediction of mortality in UBB.^25^

**Figure 6:**
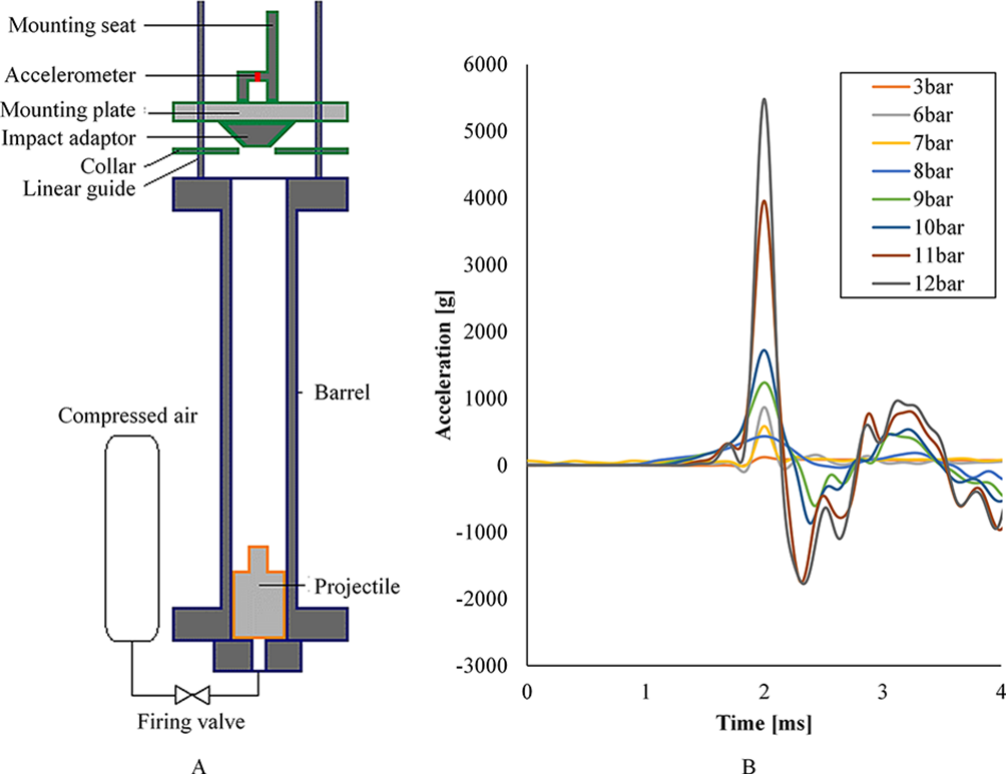
(A) Schematic of the Rig for in vivo Underbody Loading (RivUL). (B) Vertical acceleration of the RivUL seat over time in response to changing pressure input.

The Rig for in vivo Underbody Loading (RivUL) is a modified gas gun that fires a polycarbonate projectile vertically along a 1 m honed barrel (Figure 6A). The projectile is accelerated along linear rails by compressed air controlled with a solenoid valve. A seat and harness attached to the plate are used to secure anaesthetised small animals in an erect posture to simulate the seated human. The resultant axial acceleration of the seat is adjusted by altering the input pressure of the compressed air (Figure 6B).

Additional equipment, including high-speed photography, allow measurement of the biomechanical response of the animal to the loading. Injuries noted in our initial rat model include haemoperitoneum from extensive liver laceration and parenchymal lung haemorrhage. These injuries are sustained from whole-body vertical acceleration without secondary impact. Such injuries may be analogous to those described in human incidents of UBB.^25^ Further work aims to define clearly the relationship between the loading and likelihood of injury.

## Conclusion

Experimental platforms that can simulate blast loading mechanisms to the human body have been developed recently due the extensive use of explosive weapons in the last decade and the resulting need to understand the pathophysiology of blast injury. Various groups worldwide have adapted existing or created new experimental platforms to study blast injury. This paper presented a collection of platforms developed and used in CBIS to study primary, secondary and tertiary blast-injury mechanisms. The exemplar applications presented here are by no means exhaustive. Furthermore, the versatility of the designs ensures the ease of adapting the platforms to study blast injury to various parts of the body, as well as to characterise the efficacy of protective devices. For example, the shock tube can be fitted with an expansion chamber to study traumatic brain injury, where a bigger area in the sample needs to be exposed. Future adaptations of such platforms need to address the combined effects of primary and secondary blast, as injuries such as traumatic amputation or pelvic-floor rupture are a result of both primary and secondary blast mechanisms, although the contribution of each to the resulting injury is unknown.

Moreover, the well-controlled boundary conditions and injury outcomes produced from these experimental platforms offer a test bed for validation of computational simulations of blast injury. Computational simulations offer a great alternative to difficult physical experiments and a means for running a multitude of virtual tests. For example, a finite-element model of a lower-limb ATD with combat boots was developed to simulate the injuries in UBB, validated against the experiments in AnUBIS.^26^ Validated computational models can be used to understand blast injury beyond what experimentation can offer and also as design tools for protective solutions.

As the threat posed by the use of explosive devices persists, the combined efforts of an interdisciplinary research structure are required to understand blast injury fully and to contribute to the improvement in its treatment, protection and rehabilitation.
